# The E484K Substitution in a SARS-CoV-2 Spike Protein Subunit Vaccine Resulted in Limited Cross-Reactive Neutralizing Antibody Responses in Mice

**DOI:** 10.3390/v14050854

**Published:** 2022-04-21

**Authors:** Longbo Hu, Yuhua Xu, Liping Wu, Jin Feng, Lu Zhang, Yongjie Tang, Xiang Zhao, Runming Mai, Liyun Chen, Lingling Mei, Yuanzhen Tan, Yingying Du, Yanping Zhen, Wenhan Su, Tao Peng

**Affiliations:** 1 State Key Laboratory of Respiratory Disease, Sino-French Hoffmann Institute, School of Basic Medical Science, Guangzhou Medical University, Guangzhou 511436, China; longbo_hu@aliyun.com (L.H.); gdpu_wlp@163.com (L.W.); fengjin@gdscvc.com (J.F.); jj17711760868@163.com (Y.T.); zhaoxiang_789@163.com (X.Z.); 18863691261@163.com (L.M.); 2 Guangdong South China Vaccine Co., Ltd., Guangzhou 510663, China; xuyuhua@gdscvc.com (Y.X.); mairunming@gdscvc.com (R.M.); chenliyun@gdscvc.com (L.C.); tanyuanzhen2006@163.com (Y.T.); duyingying@gdscvc.com (Y.D.); zhengyanping@gdscvc.com (Y.Z.); suwenhan@gdscvc.com (W.S.); 3 Guangzhou Laboratory, No. 9 XingDaoHuanBei Road, Guangzhou International Bio Island, Guangzhou 510005, China; 4 Guangzhou Customs District Technology Center, Guangzhou 510665, China; zhanglu0307@163.com

**Keywords:** SARS-CoV-2, B.1.351, E484K, subunit vaccine, neutralizing antibody

## Abstract

Severe acute respiratory syndrome coronavirus 2 (SARS-CoV-2), especially emerging variants, poses an increased threat to global public health. The significant reduction in neutralization activity against the variants such as B.1.351 in the serum of convalescent patients and vaccinated people calls for the design of new potent vaccines targeting the emerging variant. However, since most vaccines approved and in clinical trials are based on the sequence of the original SARS-CoV-2 strain, the immunogenicity and protective efficacy of vaccines based on the B.1.351 variant remain largely unknown. In this study, we evaluated the immunogenicity, induced neutralization activity, and protective efficacy of wild-type spike protein nanoparticle (S-2P) and mutant spike protein nanoparticle (S-4M-2P) carrying characteristic mutations of B.1.351 variant in mice. Although there was no significant difference in the induction of spike-specific IgG responses in S-2P- and S-4M-2P-immunized mice, neutralizing antibodies elicited by S-4M-2P exhibited noteworthy, narrower breadth of reactivity with SARS-CoV-2 variants compared with neutralizing antibodies elicited by S-2P. Furthermore, the decrease of induced neutralizing antibody breadth at least partly resulted from the amino acid substitution at position 484. Moreover, S-4M-2P vaccination conferred insufficient protection against live SARS-CoV-2 virus infection, while S-2P vaccination gave definite protection against SARS-CoV-2 challenge in mice. Together, our study provides direct evidence that the E484K substitution in a SARS-CoV-2 subunit protein vaccine limited the cross-reactive neutralizing antibody breadth in mice and, more importantly, draws attention to the unfavorable impact of this mutation in spike protein of SARS-CoV-2 variants on the induction of potent neutralizing antibody responses.

## 1. Introduction

As of February 2022, severe acute respiratory syndrome coronavirus 2 (SARS-CoV-2), the causative agent of COVID-19, had infected more than 400 million people and led to more than 5 million deaths worldwide (https://covid19.who.int, accessed on 15 February 2022). SARS-CoV-2 is an enveloped, positive-sense, single-stranded RNA virus that belongs to the Betacoronavirus genus within the Coronaviridae family [1]. Its genome is approximately 30 kb in length and is predicted to encode 16 nonstructural proteins (nsp1–nsp16), eight accessory proteins (3a, 3b, 6, 7a, 7b, 8b, 9b, and 14), and four structural proteins (spike, membrane, envelope, and nucleocapsid) [1]. The membrane (M), spike (S), and envelope (E) proteins constitute the majority of the protein that is incorporated into the SARS-CoV-2 envelope lipid bilayer [2]. The S protein mediates attachment of the virus to host cell surface receptors and fusion between the virus and cell membranes, which is the first step in viral infection. The S protein is also the principal target of neutralizing antibodies (NAbs) generated following infection caused by SARS-CoV-2 and an important target for drug and vaccine design [3].

There are only three approved antivirals (remdesivir, paxlovid, and molnupiravir) currently available for treatment of COVID-19. Generating broadly protective vaccines against SARS-CoV-2 is an essential countermeasure to end the raging COVID-19 pandemic. However, since the outbreak of the epidemic, genetic variants of SARS-CoV-2 have been emerging and circulating around the world [3]. There is growing concern that the new SARS-CoV-2 variants that are antigenically distinct from the prototype strain render the current vaccines less effective. A variant that shows evidence of an increase in transmissibility, more severe disease, a significant reduction in neutralization by antibodies generated during previous infection or vaccination, reduced effectiveness of treatments or vaccines, or diagnostic detection failures is defined as a variant of concern (VOC). To date, there are five VOCs and their sublineages circulating worldwide, including B.1.1.7 (Alpha), first identified in the United Kingdom; B.1.351 (Beta), first identified in South Africa; P.1 (Gamma), first identified in Brazil; B.1.617.2 (Delta), first detected in India; and B.1.1.529, (Omicron) first identified in South Africa (https://www.who.int/en/activities/tracking-SARS-CoV-2-variants/, accessed on 15 February 2022). Recently, a significant reduction in neutralizing activities against the B.1.351 variant was observed in sera from individuals immunized by vaccines approved or in clinical trials, including BNT162b2 [4,5], mRNA-1273 [6], ChAdOx1-S [7], SputnikV [8], Ad26.CoV2.S [9], NVX-CoV2373 [10], BBIBP-CorV, and CoronaVac [11]. Moreover, the efficacy and effectiveness of vaccines in protecting against infection caused by the B.1.351 variant were remarkably lower than those in the context of infection caused by prototype SARS-CoV-2 or the B.1.1.7 variant infection [12,13,14]. The remarkable decrease in the neutralization activity of the B.1.351 variant in vaccinated sera occurs in part because most of the current vaccine designs were based on the sequences of the original SARS-CoV-2 strain (Wuhan-Hu-1, GenBank: MN908947.3, referred to as wild-type SARS-CoV-2), excluding the mutations in the current variants. Therefore, there is an extremely urgent need to develop novel effective vaccines for the B.1.351 variant and other emerging variants.

In addition to several approved vaccines, more than 300 candidate vaccines are currently under preclinical or clinical evaluation, including DNA vaccines, mRNA vaccines, viral vector-based vaccines, inactivated vaccines, and protein subunit vaccines [15,16]. Compared with DNA-, mRNA-, and viral-vector-based vaccines, recombinant protein subunit vaccines have proven track records of safety and efficacy in humans since they utilize specific proteins directly without transcription or translation inside the human body [17]. Recently, protein nanoparticle platforms have played an increasingly prevalent role in vaccine development, as they can present antigens at high density, facilitating the elicitation of more robust neutralizing antibodies and cellular immune responses [18]. Moreover, several protein nanoparticle vaccines displaying viral glycoprotein antigens are currently being evaluated in preclinical studies and clinical trials, such as vaccines for respiratory syncytial virus (RSV) F [19], HIV-1 envelope [20], influenza hemagglutinin [21], and SARS-CoV-2 spike [13,18,22,23].

Here, we generated and compared two designed protein nanoparticle vaccines displaying a stabilized prefusion wild-type SARS-CoV-2 S protein (S-2P) or mutant S protein (S-4M-2P) carrying four defining mutations of the B.1.351 variant. Surprisingly, S-4M-2P immunization induced only a limited neutralization response and offered relatively poor protection in hACE2 mice challenged with the live SARS-CoV-2 virus compared with S-2P immunization, resulting at least partly from immunogenicity or conformational changes caused by mutations at site 484. To our knowledge, there are very few reports comparing the immunogenicity and protection of prototype S protein nanoparticle vaccines with mutant S protein carrying characteristic mutations of the B.1.351 variant. Our results suggest that the prototype S protein nanoparticle vaccine elicits a much broader neutralization response than mutant S carrying the E484K mutation, further highlighting that the effects of site 484 mutations should be considered more carefully when designing new vaccines based on SARS-CoV-2 variants carrying E484K mutation.

## 2. Materials and Methods

### 2.1. Cell Lines

Sf-βX, a rhabdovirus-negative Sf9 cell line derived from SF-α cells (Southern China United Vaccine Institute, Guangzhou, China), was maintained in serum-free medium (Vigor-S100S, BIOENGINE, Saffron Walden, UK) at 27 °C. HEK293T cells were maintained in DMEM (GIBCO, Waltham, MA, USA) supplemented with 10% fetal bovine serum (FBS, Gibco, Waltham, MA, USA), 100 mg/mL streptomycin, and 100 unit/mL penicillin at 37 °C in 5% CO_2_. The 293T-hACE2 cell line stably expressing human angiotensin-converting enzyme 2 (ACE2) was generated by lentiviral transduction of human ACE2 into HEK293T cells, followed by stable cell selection with puromycin.

### 2.2. Cloning, Protein Expression and Purification of SARS-CoV-2 Spike Protein

The codon-optimized SARS-CoV-2 spike gene encoding residues 1–1252 of the spike protein of the Wuhan-Hu-1 strain (GenBank: MN908947) was synthesized and inserted into the pOET1 vector to generate the WT-S construct. The S-2P construct was made through two prolines introduced at residues 986 and 987 (986-KV-987 to 986-PP-987) to stabilize the prefusion conformation and “QQAQ” substituted at the furin cleavage site (682-RRAR-685 to 682-QQAQ-685) to confer protease resistance. Four key mutations in the B.1.351 variant, namely K417N, E484K, N501Y, and D614G, were simultaneously introduced into S-2P to obtain the S-4M-2P construct. Recombinant baculoviruses expressing the S-2P or S-4M-2P proteins were generated using the flashBAC ULTRA system (Oxford Expression Technologies, Oxford, UK) following the manufacturer’s instructions. Sf-βX cells were cultured in serum-free medium (Vigor-S100S, BIOENGINE) and infected with recombinant baculovirus at an MOI = 0.5 in a ReadyToProcess WAVE 25 Rockor (GE Healthcare Life Sciences, Marlborough, MA, USA) at 27 °C. Cells were harvested by centrifugation at 72 h post infection and then suspended in 25 mM Tris HCl (pH 8.0), 50 mM NaCl, and 0.5–1.0% (*v*/*v*) TERGITOL NP-9. The supernatant containing recombinant protein S-2P or S-4M-2P was clarified by centrifugation at 10,000× *g* for 30 min, followed by subsequent purification with TMAE anion exchange and lentil lectin affinity chromatography. Purified spike protein was formulated in 25 mM sodium phosphate (pH 7.2), 300 mM NaCl, and 0.02% (*v*/*v*) polysorbate 80 (PS 80) by a tangential flow ultrafiltration system and then subjected to SDS-PAGE and Western blot analyses.

### 2.3. Production of Pseudotyped Lentiviral Particles with Wild-Type and Variant SARS-CoV-2 Spike Proteins

The truncated SARS-CoV-2 spike gene of the Wuhan-Hu-1 strain (GenBank: MN908947) with a C-terminal 21 aa deletion was codon-optimized for mammalian cell expression and inserted into the eukaryotic expression vector pcDNA3.1 to obtain the construct pcDNA3.1-nCoV-S. A series of additional mutations were introduced into pcDNA3.1-nCoV-S to generate constructs containing the variant spike proteins of B.1.1.7 (69del, 70del, 144del, N501Y, A570D, D614G, P681H, T716I, S982A, and D1118H); B.1.351 (D80A, D215G, 241del, 242del, 243del, K417N, E484K, N501Y, D614G, and A701V); P.1 (L18F, T20N, P26S, D138Y, R190S, K417T, E484K, N501Y, D614G, H655Y, and T1027I); B.1.617.2 (T19R, T95I, G142D, 156del, 157del, R158G, L452R, T478K, D614G, P681R, and D950N); and other variants, as illustrated in Figure 4A. HEK293T cells were co-transfected with plasmid (pcDNA3.1) expressing wild-type or variant SARS-CoV-2 spike protein, lentiviral vector (pWPXL) expressing firefly luciferase reporter protein, and lentiviral packaging plasmid (psPAX2) at a ratio of 6:2:3 using polyethylenimine (PEI MAX, Polysciences, Inc., Warrington, PA, USA). The medium was refreshed one hour prior to transfection and 12 h post transfection. Supernatants containing pseudotyped lentiviral particles were harvested at 48 h post transfection, filtered through a 0.45 μm low-protein binding filter, and then kept at −80 °C.

### 2.4. SARS-CoV-2 Pseudovirus Titration

The titration of the SARS-CoV-2 pseudovirus was performed as described previously [24], with some modifications. Briefly, 293T-hACE2 cells (3.5 × 10^5^ cells/mL) were seeded in 96-well plates one day before virus infection and then infected with a series of 3-fold dilutions of SARS-CoV-2 pseudovirus (6 wells per dilution factor). Cells without the addition of pseudovirus were used as controls. After 48 h of incubation, the culture supernatant was aspirated gently, and the cells were lysed for luminescence detection using a Bright-Glo Luciferase Assay (Promega, Dane County, WI, USA), according to the manufacturer’s instructions, in a microplate luminometer (GloMax, Promega). A signal with ten-fold relative luminescence unit (RLU) values higher than those of the control was determined to be positive. The 50% tissue culture infectious dose (TCID_50_) was calculated using the Reed–Muench method.

### 2.5. SARS-CoV-2 Pseudovirus-Based Neutralization Assay

The pseudovirus-based neutralization assays were performed as reported [25] with minor modifications. In brief, the heat-inactivated serum samples were initially diluted at 1:50 (*v*/*v*) and then mixed with 250 TCID_50_ SARS-CoV-2 pseudovirus at 1:1 (*v*/*v*). After incubation for 30 min at 37 °C, the mixture was added to 293T-hACE2 cells in a 5% CO_2_ 37 °C incubator. Forty-eight hours later, cell lysates were collected for luminescence detection using a Bright-Glo Luciferase Assay (Promega) following the manufacturer’s protocol in a microplate luminometer (GloMax, Promega). Neutralization curves were made with GraphPad Prism (GraphPad Software, Inc., La Jolla, CA, USA), and the ID_50_ (50% inhibitory dilution neutralization titers) was calculated using a nonlinear regression (log(inhibitor) versus normalized response − variable slope) algorithm.

### 2.6. Mouse Immunization

Mouse immunization experiments were performed in Foshan Huamiao Biotechnology Company Ltd. (Foshan, China) Wild-type BALB/c mice were injected intramuscularly with 0.2 or 1 μg SARS-CoV-2 spike protein twice on day 0 (prime) and 14 (boost) adjuvanted with aluminum hydroxide (*n* = 8 for each dosage). A placebo group (*n* = 8) with adjuvant alone served as a nonimmunized control. Serum was collected for analysis before the initial immunization and at the indicated time points after the final immunization.

### 2.7. Mouse Challenge Experiments

hACE2 transgenic mice (6 weeks old, C57BL/6-Tgtn, CAG-human ACE2-IRES-Luciferase-WPRE-polyA) were provided by Shanghai Model Organisms (Shanghai, China). Mouse challenge experiments were performed in the Biosafety Level 3 (BSL3) Laboratories of Guangzhou Customs District Technology Center. hACE-2 mice were vaccinated through intramuscular injection with 1 or 5 μg SARS-CoV-2 spike protein twice at day 0 and day 14 adjuvanted with aluminum hydroxide (*n* = 10 for each dosage). A placebo group vaccinated with only adjuvant served as a nonvaccinated control (*n* = 10). Boost sera were collected 2 weeks after the final immunization. Vaccinated and control animals were intranasally challenged with 1 × 10^5^ pfu of live SARS-CoV-2 virus (GenBank: MT123290) 16 days after the final immunizations. At 3 days post infection, mice were sacrificed. Lung tissues were collected to examine viral RNA levels or fixed with 10% formalin for histological examination using hematoxylin and eosin (H&E) staining.

### 2.8. Enzyme-Linked Immunosorbent Assay (ELISA)

SARS-CoV-2 spike protein-specific IgG was analyzed by ELISA. Briefly, 96-well microtiter plates were coated with 1.0 µg /mL of target protein overnight at 4 °C. Plates were washed with phosphate-buffered saline with 0.1% Tween 20 (PBST) and blocked for 2 h using 200 μL of 5% nonfat milk. Mouse serum samples were serially diluted twofold and added to the blocked plates, followed by a 2 h incubation at 37 °C. Plates were washed with PBST and incubated with HRP-conjugated goat anti-mouse IgG (Sigma Aldrich, MI, USA) at a 1:10,000 dilution. After incubation for another 1 h at room temperature, the plates were washed with PBST and developed with TMB/E substrate (Merck Millipore, Burlington, MA, USA). Reactions were stopped with 1 M H_2_SO_4_, and the optical density at 450 nm (OD_450_) values were read with an Epoch microplate spectrophotometer (BioTek Instruments Inc., Winooski, VT, USA). All raw OD_450_ values had blank values subtracted before analysis. A subtracted OD_450_ value above 0.1 and two-fold greater than that of the no-serum control was considered positive, and the maximum dilution with a positive result was used as the ELISA titer.

### 2.9. Electron Microscopy (EM)

Electron microscopy was performed using a Tecnai G2 Spirit transmission electron microscope operated at 120 kV. SARS-CoV-2 S protein samples were applied to nitrocellulose-supported 400-mesh copper grids and stained with uranyl formate. High-magnification images were acquired with an FEI Eagle 4 k × 4 k CCD camera.

### 2.10. Statistical Analysis

One-way ANOVA followed by Dunnett’s multiple comparisons test was used for statistical analysis (GraphPad Prism 8). *p* < 0.05 was considered statistically significant. (* *p* < 0.05, ** *p* < 0.01, *** *p* < 0.001, **** *p* < 0.0001).

## 3. Results

### 3.1. Construction and Characterization of SARS-CoV-2 Spike Protein Nanoparticle Vaccines

**Figure 1:**
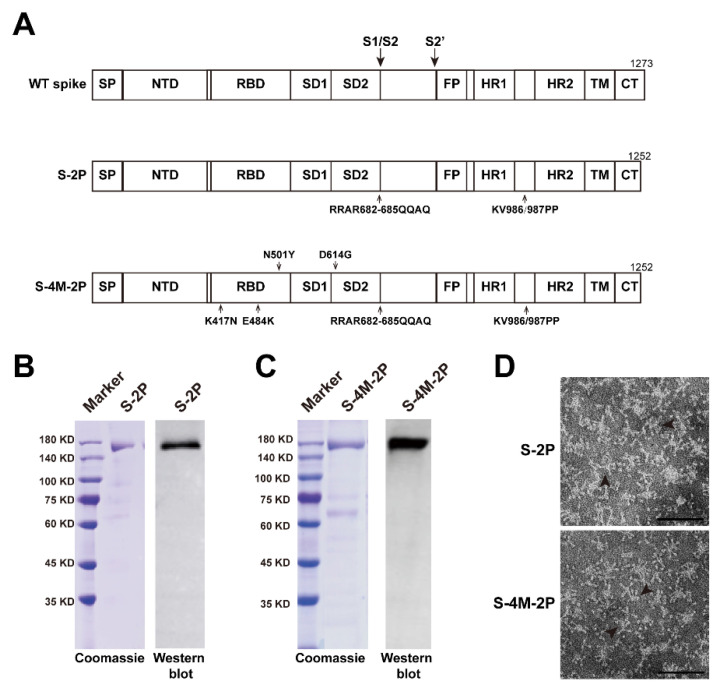
Preparation of SARS-CoV-2 spike protein nanoparticle. (**A**) Schematic representation of SARS-CoV-2 spike (WT spike) and two prefusion-stabilized and furin-cleavage-deficient constructs (S-2P and S-4M-2P). Amino acid substitutions are indicated by arrows. SP, signal peptide; NTD, N-terminal domain; RBD, receptor-binding domain; SD1, subdomain 1; SD2, subdomain 2; S1/S2, protease cleavage site; S2′, protease cleavage site; FP, fusion peptide; HR1, heptad repeat 1; HR2, heptad repeat 2; TM, transmembrane domain; CT, C-terminal tail. The spike protein S-2P (**B**) and S-4M-2P (**C**) expressed in SF9 cells was purified and analyzed by Coomassie-stained SDS-PAGE (**left**) and Western blot (**right**). Molecular weight standards are indicated on the left in kDa. (**D**) Representative negative-stain electron micrographs for S-2P (**upper**) and S-4M-2P (**lower**) nanoparticles (arrowhead), scale bar = 100 nm.

The neutralization activity of sera from convalescents or individuals immunized with many approved vaccines against the B.1.351 variant decreased significantly [3]. Thus, it is extremely urgent to enhance protection against B.1.351 variant infection. To date, most current authorized or approved vaccines have been developed based on the wild-type SARS-CoV-2 (Wuhan-Hu-1, GenBank: MN908947.3) sequence, whose immunogenicity and protective effect of vaccines based on SARS-CoV-2 variants sequences remain unknown. Therefore, we designed two vaccines displaying the wild-type S protein or mutant S protein carrying the featured mutations of the B.1.351 variant. The coding sequence of the original SARS-CoV-2 strain (Wuhan-Hu-1, GenBank: MN908947.3) S protein (WT spike) was codon-optimized for expression in insect cells. S-2P was generated by introducing two proline substitutions at residues 986 and 987 as well as QQAQ substations between residues 682 and 685 of the WT spike. Substitutions of 986-KV-987 to 986-PP-987 (2P) were introduced to stabilize the prefusion conformation of spike protein [26,27,28,29,30], and modifications at the furin cleavage site (682-RRAR-685 to 682-QQAQ-685) were introduced to protect S-2P from protease digestion [31,32]. S-4M-2P was constructed by further introducing four featured mutations (K417N, N501Y, E484K, and D614G) of the B.1.351 variant into S-2P simultaneously (Figure 1A). Both S-2P and S-4M-2P were cloned into recombinant baculovirus, expressed in Sf-βX cells, and verified by SDS–PAGE and Western blotting after further purification. The purified S-2P and S-4M-2P proteins had a molecular weight of 170 kDa (Figure 1B,C). Transmission electron microscopy (TEM) showed that in the presence of polysorbate 80, both S-2P and S-4M-2P formed nanoparticles with uniform size and shape (Figure 1D).

### 3.2. S-2P Induced a Broader Spectrum of Neutralizing Responses Than S-4M-2P

**Figure 2:**
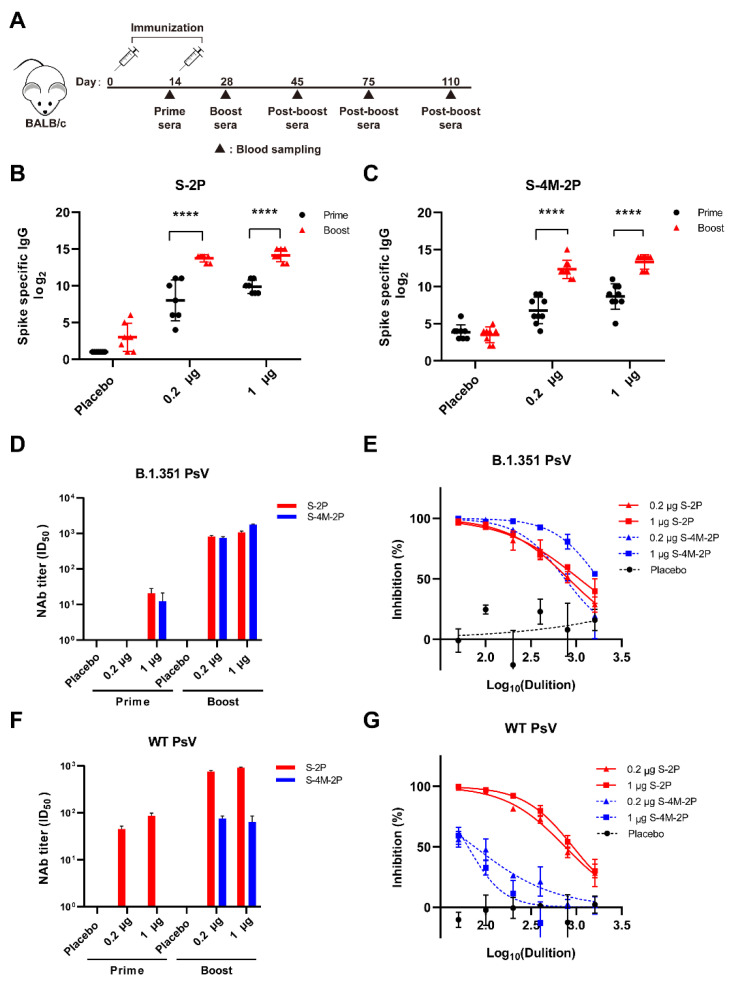
Immunogenicity of the WT SARS-CoV-2 and variant spike proteins in mice. The prime-boost regimen using spike proteins adjuvanted with aluminum hydroxide results in sera possessing neutralization activity. (**A**) Schematic diagram of prime-boost vaccination strategy and sample collection schedule. BALB/c mice were prime immunized i.m. with low-dose (0.2 μg, *n* = 8) or high-dose (1 μg, *n* = 8) spike proteins adjuvanted with aluminum hydroxide or adjuvant only (placebo group, *n* = 8) at day 0 and boosted with an equivalent dose 14 days later. Sera were collected at the indicated time points for further assays. SARS-CoV-2 spike-specific IgG titers of sera from S-2P-immunized mice (**B**) and S-4M-2P-immunized mice (**C**) were determined by ELISA. The sera from each group collected at the same time were mixed together in equal quantities, and pseudovirus-based neutralization assays were performed to detect neutralizing antibody (NAb) titers (ID_50_) of prime and boost sera from S-2P- or S-4M-2P-immunized mice against WT SARS-CoV-2 spike (**D**) or B.1.351 variant spike (**E**) pseudovirus. Data are shown as mean ± SEM, representative of two independent experiments with two replicates. Representative neutralization curves of boost sera from S-2P- or S-4M-2P-immunized mice against WT SARS-CoV-2 spike (**F**) or B.1.351 variant spike (**G**) pseudovirus. PsV, pseudovirus. The log_10_-transformed dilutions of the sera are shown against the percentage of neutralization. **** *p* < 0.0001.

Then, the immunogenicity of S-2P and S-4M-2P was assessed in mice. BALB/c mice were immunized with low doses (0.2 μg) or high doses (1 μg) of S-2P and S-4M-2P with aluminum hydroxide adjuvant using a prime/boost regimen spaced 14 days apart (Figure 2A). Mice immunized with S-2P or S-4M-2P had high S-specific IgG titers following the prime immunization and significantly elevated S-specific IgG titers after the boost immunization (Figure 2B,C). The neutralization titers of these vaccinated sera against WT and variant SAR-CoV-2 were determined using in vitro pseudovirus-based neutralization assays. Animals immunized with S-2P or S-4M-2P displayed comparable neutralization titers against B.1.351 pseudoviruses after the boost immunization (Figure 2D,E). However, the neutralization titers against wild-type SARS-CoV-2 pseudoviruses were significantly lower in S-4M-2P-immunized animals than in S-2P-immunized animals (Figure 2F,G).

### 3.3. NAbs Induced by S-4M-2P Mostly Recognized Pseudoviruses Carrying Lys at Site 484 in Spike Protein

**Figure 3:**
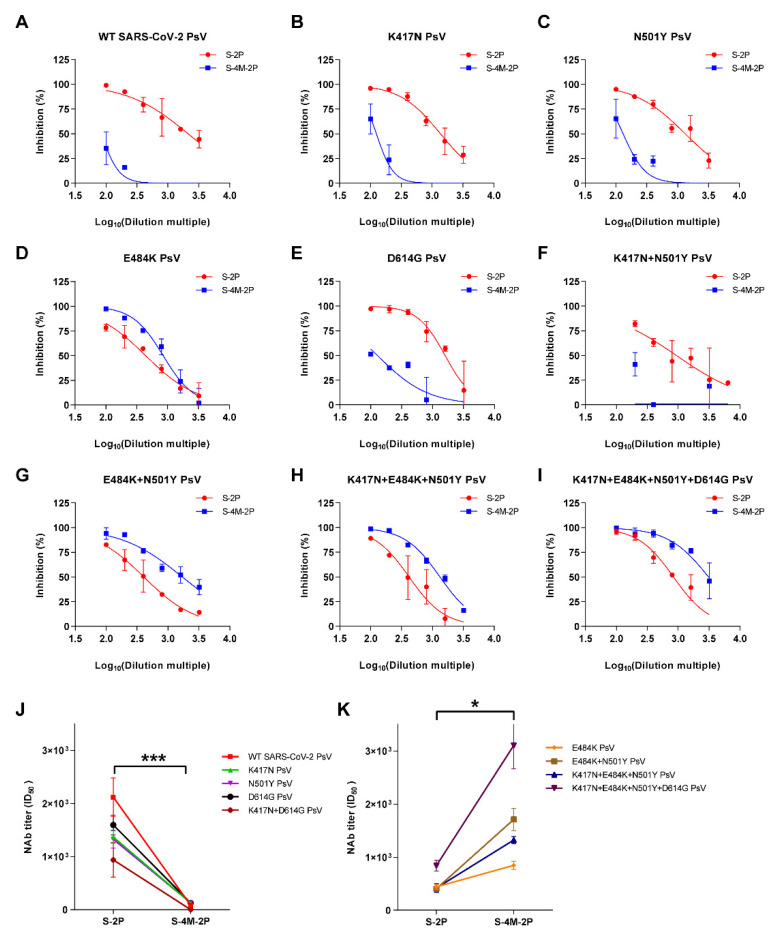
Neutralizing antibodies elicited by S-4M-2P mostly targeted the mutant residue K484 but not the wild-type residue E484. Boost sera from the high-dose (1 µg) vaccinated mice group were collected at 28 days after initial vaccination and mixed together in equal quantities. Pseudovirus-based neutralization assays were performed to detect neutralizing antibody titers (ID_50_) of sera against pseudotyped virus carrying wild-type spike or spike with single or multiple mutations. Representative neutralization curves of boost sera from mice vaccinated with 1 μg S-2P or S-4M-2P protein against pseudotyped virus carrying wild-type SARS-CoV-2 spike (**A**) or spike carrying the K417N (**B**), N501Y (**C**), E484K (**D**), D614G (**E**), K417N+N501Y (**F**), E484K+N501Y (**G**), K417N+E484K+N501Y (**H**), and K417N+E484K+N501Y+D614G (**I**) mutations. The log_10_-transformed dilutions of the sera are shown against the percentage of neutralization. Neutralization titer (ID_50_) of boost sera from S-2P- or S-4M-2P-vaccinated mice against pseudotyped virus carrying SARS-CoV-2 spike without E484K mutant (**J**) or with E484K mutant (**K**). Data are shown as mean ± SEM, representative of two independent experiments with two technical replicates. * *p* < 0.05, *** *p* < 0.001.

To investigate the impact of mutations in the spike protein on neutralizing responses, we constructed a panel of eight SARS-CoV-2 pseudoviruses, including four single mutations and four combined mutations, based on four key mutations of B.1.351 spike protein (K417N, D501Y, E484K, and D614G) in S-4M-2P. Then, we performed neutralization tests using sera from S-2P- or S-4M-2P-immunized animals (Figure 3A–I). The results showed that S-4M-2P induced significantly lower neutralization activity than S-2P against pseudoviruses without the E484K mutation (Figure 3J) and, by contrast, obviously higher neutralization activity against pseudoviruses carrying E484K (Figure 3K). Our results indicated that S-4M-2P-induced neutralizing antibodies mostly neutralized pseudotyped viruses carrying the E484K mutation, suggesting that the E484K mutation in S-4M-2P was the prime determinant for the induction and recognition of neutralizing antibodies.

**Figure 4:**
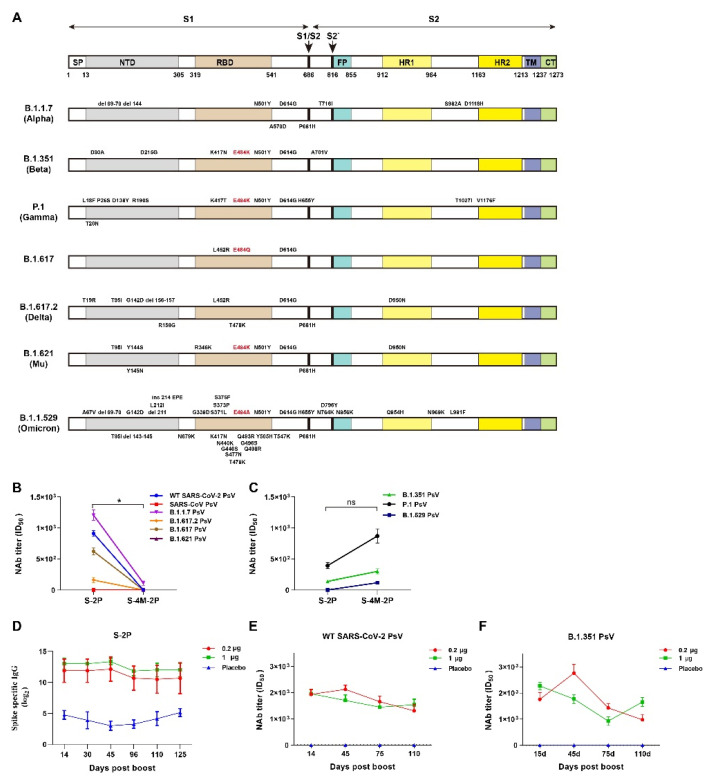
Neutralizing antibodies elicited by S-4M-2P mostly targeted the variants carrying E484K mutation. (**A**) Schematic illustration of structural components of spike protein of SARS-CoV-2 and respective mutations in the spike protein of B.1.1.7, B.1.351, P.1, B.1.617, B.1.617.2, B.1.621, and B.1.1.529 SARS-CoV-2 variants. Mutations at site 484 are marked in red. Neutralization titer (ID_50_) of boost sera from S-2P- or S-4M-2P-vaccinated mice against SARS-CoV, wild-type, and SARS-CoV-2 variant pseudotyped viruses without E484K mutation (**B**) or with E484K mutation (**C**). Data are shown as mean ± SEM, representative of two independent experiments with 2 replicates. (**D**) Sera from S-2P-immunized mice were collected at the indicated time points, and SARS-CoV-2 spike-specific IgG titers were determined by ELISA. Sera from the S-2P-immunized mouse group were collected at the indicated time points and mixed together in equal quantities, and neutralization titers (ID_50_) against WT SARS-CoV-2 (**E**) or B.1.351 variant (**F**) pseudovirus were assayed. Data are shown as mean ± SEM, representative of two independent experiments with two replicates. * *p* < 0.05, ns: not significant.

Moreover, different mutations at site 484 appeared in SARS-CoV-2 variants, such as E484Q in B.1.617 variant, E484A in B.1.1.529 variant, and E484K in B.1.351, P.1, and B.1.621 variants (Figure 4A). To evaluate the ability of vaccine-induced neutralizing antibodies to recognize different amino acids at site 484, we compared the neutralizing activity of the serum from S-2P- or S-4M-2P-nanoparticles-immunized mice against SARS-CoV and SARS-CoV-2 variant pseudoviruses. Specifically, compared with S-4M-2P-nanoparticle-vaccine-induced antibody in mouse sera, S-2P-nanoparticle-vaccine-induced antibody exhibited significantly greater neutralizing activity against B.1.1.7, B.1.617, and B.1.617.2 variants and wild-typed SARS-CoV-2 pseudoviruses, which carry original glutamic acid or mutated glutarnine at 484 site, and slight weaker neutralizing activity against B.1.351, P.1, and B.1.519 variants pseudoviruses, which carry mutated Lysine at 484 site (Figure 4B,C). Neither S-2P nor S-4M-2P nanoparticle elicited detectable neutralization activity against the SARS-CoV and B.1.1.529 variant pseudoviruses (Figure 4B). Our results indicated that S-4M-2P-elicited neutralizing antibodies only recognized SARS-CoV-2 pseudoviruses carrying E484K mutation, while S-2P induced a broader spectrum of neutralizing responses than S-4M-2P.

Previous studies have shown that the titers of neutralizing antibody in sera from convalescent and vaccinated individuals gradually decline over time [33,34,35]. We wondered whether the titers of neutralizing antibodies in S-2P-immunized animals have a similar tendency to decline over time and hence measured the S-specific IgG and neutralizing antibody titers at different time points after boost immunization. The results showed no evident change in the level of S-specific IgG from 14 to 125 days after boost immunization (Figure 4D). Although the neutralizing antibody titers against wild-type SARS-CoV-2 pseudoviruses slowly declined over time, they remained comparatively high even at 110 days after S-2P boost immunization (Figure 4E,F). These results suggested that S-2P immunization could confer long-lasting protection.

### 3.4. S-2P Elicited Stronger Protective Immune Responses against SARS-CoV-2 Infection Than S-4M-2P in Mice

**Figure 5:**
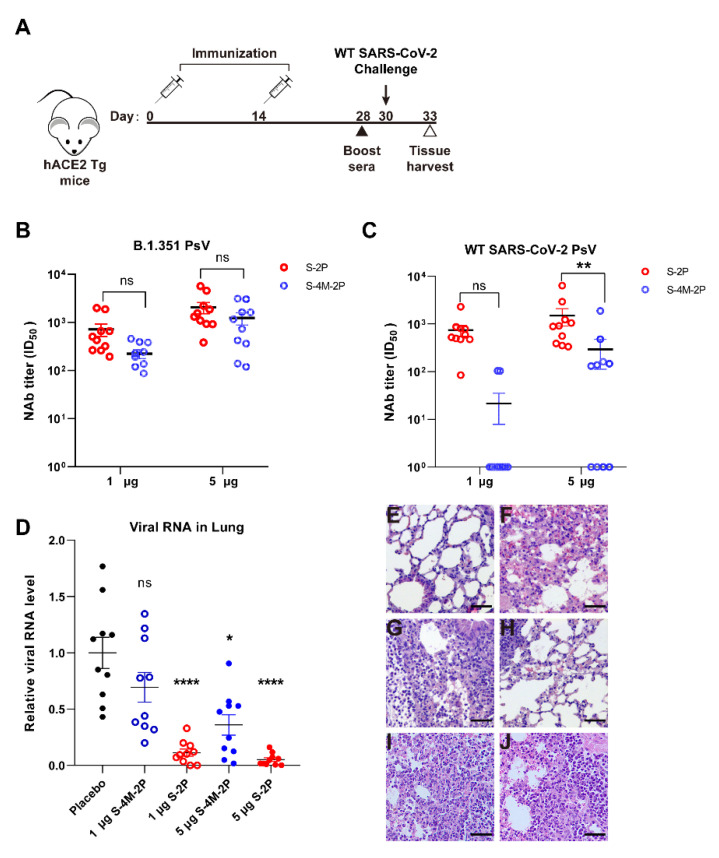
Protective efficacy of SARS-CoV-2 spike proteins against wild-type SARS-CoV-2 challenge in hACE2 transgenic mice. Groups of hACE2 transgenic mice (*n* = 10) received two doses of 1 μg or 5 μg spike protein nanoparticle adjuvanted with aluminum hydroxide or adjuvant only via the i.m. route. Mice were challenged with 1 × 10^5^ FFU of live SARS-CoV-2 virus at 30 days post initial vaccination. (**A**) Schematic diagram of mouse immunization and challenge schedule. Boost sera collected at 4 weeks after the initial S-2P or S-4M-2P vaccination were examined for neutralizing antibody titers against B.1.351 variant (**B**) and wild-type SARS-CoV-2 (**C**) pseudotyped virus. (**D**) SARS-CoV-2 RNA levels in the lungs of mice immunized with S-2P or S-4M-2P were determined by RT-PCR. Pathologic analysis of hematoxylin and eosin-stained lung tissue sections from hACE2 transgenic mice after no treatment (**E**), immunized with adjuvant only (**F**), or immunized with 1 μg S-2P (**G**), or 5 μg S-2P (**H**), or 1 μg S-4M-2P (**I**), or 5 μg S-4M-2P (**J**) nanoparticle at 3 days post SARS-CoV-2 infection. Scale bar = 50 μm. * *p* < 0.05, ** *p* < 0.01, **** *p* < 0.0001, ns: not significant.

To further evaluate the potential efficacy of nanoparticle vaccines, hACE2 transgenic mice were challenged with live SARS-CoV-2 (Wuhan-Hu-1) intranasally 16 days after S-2P or S-4M-2P boost immunization (Figure 5A). Consistent with previous results, S-2P elicited comparable neutralizing antibody titers against wild-type SARS-CoV-2 and B.1.351 variant pseudotyped virus in mice immunized with a high dose, whereas S-4M-2P induced neutralizing antibodies against only B.1.351 pseudovirus and hardly elicited neutralizing antibodies against wild-type SARS-CoV-2 pseudovirus even in mice immunized with a high dose (Figure 5B,C). We subsequently measured genomic RNA loads in lungs of SARS-CoV-2-infected mice by RT-qPCR. For mice receiving either a low dose or high dose of S-2P vaccine, we observed noteworthy decreased levels of viral RNA in the lung in comparison with levels seen in the placebo control mice (Figure 5D). For S-4M-2P, however, obviously declined levels of viral RNA were only observed in the lungs of mice immunized with high-dose but not low-dose S-4M-2P vaccine (Figure 5D).

Lung tissues were also collected at 3 days post challenge, and sections were stained with hematoxylin and eosin for pathologic analysis. In sharp contrast to the lungs of placebo control animals (Figure 5F), which were injured and characterized by infiltration of inflammatory cells, protein exudation, and thickened alveolar septa, the lungs of mice immunized with high-dose S-2P were relatively healthy, with little inflammation (Figure 5G,H). However, the lungs of S-4M-2P-immunized mice displayed injury features similar to those of placebo control mice (Figure 5I,J). Collectively, these data suggest that immunization with S-2P is able to generate a protective immune response, alleviate SARS-CoV-2-induced lung damage, and protect against SARS-CoV-2 infection in mice.

## 4. Discussion

The most cost-effective and potentially long-term solution to the fight against a viral disease is to develop highly efficacious vaccines. Recent SARS-CoV-2 vaccine candidates are classified into five types: DNA vaccines, mRNA vaccines, virus vectored vaccines, inactivated vaccines, and protein subunit vaccines [36]. Protein subunit vaccines have a proven safety record in humans but are usually not sufficiently immunogenic. Generally, there are two major approaches to improve subunit vaccine immunogenicity for the production of high levels of neutralizing antibodies. One approach is to optimize vaccine design, such as the structure-based antigen design used in this study. Structure-based vaccine design seeks to create more efficient immunogens that will elicit protective antibody responses against the target pathogen based on a progressive understanding of antigen structure and function [37]. Due to the successful application of structure-based designed immunogens to induce the desired immune responses against RSV [38,39], HIV [40,41], PIV [42], and HCV [43,44], we designed a SARS-CoV-2 spike subunit vaccine based on the current understanding of the structures and fusion mechanisms of class I viral fusion proteins, such as RSV F [45], coronavirus S [46], and influenza HA protein [47]. Specifically, we introduced two proline substitutes to stabilize the prefusion conformation of the spike protein: a mutant furin cleavage site to protect the spike protein from protease digestion and a truncated cytoplasmic tail to increase spike protein expression on the cell surface [48]. Potent and broad-spectrum NAbs elicited by S-2P nanoparticle immunization and protective effects against live original SARS-CoV-2 virus infection suggested the great potential of the vaccine we designed. Regrettably, the protective effect of S-2P or S-4M-2P immunization against live B.1.351 variant infection has not been tested because of the inaccessibility of live B.1.351 variant virus. However, S-2P-induced broad-spectrum NAbs against SARV-CoV-2 variants pseudovirus at least suggest that this vaccine has the potential to protect infections caused by multiple variants.

Another approach to improve subunit vaccine immunogenicity is the addition of effective adjuvants. We noticed that despite the very low viral RNA load, viral RNA could still be detected in the lungs of mice inoculated with the S-2P vaccine (Figure 5D), highlighting that there is still room for improvement in the protective effect. We speculate that the inability to completely prevent the infection was at least partly due to the insufficient efficiency of the aluminum adjuvant used in this study. Although aluminum adjuvant is the most frequently used adjuvant in human vaccines, it usually results in polarization toward a Th2 response, which has been regarded as unfavorable in the case of coronavirus and other viral infections and vaccinations [17,49]. Recently, several vaccine adjuvants have shown great potential in coronavirus vaccine strategies but are still in development, including MF59, AS03, Addavax, CpG ODN, and Matrix-M [17]. The effect of the S-2P vaccine in this study combined with these new adjuvants is promising.

Amino acid position 484 is located in the interaction interface with ACE2 of the receptor binding domain (RBD) of the spike protein and is involved in binding with ACE2 [50]. The E484K mutation first appeared in March 2020 and then rapidly spread and became one of the shared defining mutations of many SARS-CoV-2 variants, including the B.1.351 (Beta), P.1 (Gamma), P.2 (Zeta), P.3 (Theta), B.1.621 (Mu), B.1.526 (Lota), and B.1.525 (Eta) variants (https://covariants.org, accessed on 15 February 2022). Recent studies have suggested that the E484K mutation increases ACE2 affinity [51] and accounts for immune escape of B.1.351 and P.1 due to the reduction in neutralizing antibody recognition caused by conformational changes [7,52,53,54,55]. However, the effect of the E484K mutation on immunogenicity and neutralization breadth remains unknown. Our results indicated that more potent and cross-reactive neutralizing responses were induced by the wild-type spike protein than by the spike protein carrying the E484K mutation. The NAbs elicited by spike protein carrying the E484K mutation exclusively recognized SARS-CoV-2 pseudoviruses with Lys at 484 site of spike protein but not recognized SARS-CoV-2 pseudoviruses with Glu, Ala, or Gln at 484 site of spike protein (Figure 4B,C). The limited neutralization breadth and potency resulting from the E484K mutation raised concern about the protection of designed vaccines based on the SARS-CoV-2 variants carrying E484K mutation in spike protein against infection with the emerging SARS-CoV-2 lineages without E484K mutation. As expected, S-4M-2P vaccination offered relatively poor protection in hACE2 mice challenged with the original SARS-CoV-2. Of note, our findings are consistent with recent studies that have suggested that plasma from B.1.351 variant-infected patients displayed remarkably lower neutralization of the original variant than of the B.1.351 variant [56,57]. Substitution of negatively charged residue Glu484 by Lys with positive charge changed the electrostatic energies and in turn contributed to the increased binding affinity with hACE2 as well as reduced interactions with neutralizing antibodies [58,59]. We speculate that the change of electrostatic energies caused by E484K mutation is at least partly responsible for the narrow breadth of the neutralizing antibody. The underlying mechanisms, however, need further investigation.

To counteract the impact of circulating variants, one option is to develop new vaccines that more closely reflect the variants. However, it is not clear how beneficial such vaccines designed specifically to target new variants will be. Although the underlying mechanism is not entirely clear, our findings provide the first preliminary evidence that the E484K mutation results in poor cross-reactivity of NAbs except for resulting in immune escape by affecting the recognition of neutralizing antibodies. Consequently, careful consideration should be given when designing new vaccines based on SARS-CoV-2 variants, especially variants with the E484K mutation in spike protein, to potentiate vaccine efficacy.

## Data Availability

The datasets generated during and/or analyzed during the current study are available from the corresponding author on reasonable request.
